# Brown seaweed: *Fucus vesiculosus* as a feedstock for agriculture and environment protection

**DOI:** 10.1038/s41598-023-36881-z

**Published:** 2023-06-21

**Authors:** Karolina Krautforst, Anna Szymczycha-Madeja, Maja Wełna, Izabela Michalak

**Affiliations:** 1 Department of Advanced Material Technologies, Faculty of Chemistry, Wrocław University of Science and Technology, Smoluchowskiego 25, 50-372 Wrocław, Poland; 2 Department of Analytical Chemistry and Chemical Metallurgy, Faculty of Chemistry, Wrocław University of Science and Technology, Wybrzeże Wyspiańskiego 27, 50-370 Wrocław, Poland

**Keywords:** Mass spectrometry, Environmental biotechnology, Analytical chemistry, Chemical engineering, Environmental chemistry, Biological techniques, Biotechnology, Environmental sciences

## Abstract

A comprehensive approach to the management of brown seaweed—*Fucus vesiculosus* was presented. An algal extract, which served as a biostimulant of plant growth was produced using ultrasound-assisted extraction (UAE). The concentration of the extract (20, 40, 60, 80, 100%), which had the greatest influence on biometric parameters of radish, was determined in germination tests. The seaweed itself as well as the produced post-extraction residue were used in doses of 2 and 4 g/kg as soil additives, stimulating plant growth in the initial phase. Pot tests for sorghum carried out under optimal conditions (20% extract and 2 g/kg of soil additive) had a positive effect on the plant weight, length and the content of chlorophyll in comparison with the control group treated with distilled water. Additionally, preliminary studies on the bioremediation of soil contaminated with Zn(II) ions with the use of both soil additives were performed. It was shown that the immobilization of Zn(II) ions in the soil by the applied additives reduced the bioaccumulation of zinc in the aerial part of plants as compared with the group cultivated in the contaminated soil but without additive. Accordingly, by producing plant biostimulants by UAE it was also possible to successfully manage the post-extraction residue following the concept of a bio-based economy.

## Introduction

Currently, a growing problem in agriculture is the lack of access to land for cultivation as well as the increasing resistance of weeds, pathogens and pests to the previously used plant protection products. Greater susceptibility to pathogens as well as changing weather conditions are one of the main reasons for lowering the quality and size of the crop^1^. Additionally, as a result of intensively developing industry and public transport along with inadequately used mineral fertilizers and pesticides, the soil is more and more often contaminated with toxic metals, making it unsuitable for plant cultivation^2^. Therefore, in order to feed the entire human population in the future, it is necessary to strive to increase production with reduced arable land. The growth in the efficiency and productivity of agriculture should be associated with the maintenance or increase in plant resistance to the biotic and abiotic stress^3^. The solution to the above-mentioned problems may be the use of natural products obtained from biomass. Marine algae (seaweeds), which constitute waste in many regions all over the world and should be managed following the principles of sustainable development can be a source of easily available biomass^4^. This raw material is very valuable due to its unique chemical composition^5^. Seaweed biomass contains polysaccharides (e.g., fucoidan, agar, algin, or laminarin), lipids (including polyunsaturated fatty acids), amino acids, natural pigments, macro- (e.g., K, Ca, Mg) and microelements (e.g., Fe, Cr, Mn), vitamins such as A, B, C or E, as well as compounds with antioxidant activity such as polyphenols^6–9^.

Seaweeds and products derived therefrom can be used as biostimulants of plant growth (usually applied in form of extracts), stimulating plant growth processes and improving stress resistance by increasing the efficiency of nutrients uptake and utilization^10^. On the other hand, they can be used as biosorbents in the bioremediation of soil contaminated with heavy metals^11–14^. The process of metal immobilization in soil using additives in organic or inorganic form reduces their mobility and bioavailability to plants^15,16^.

Although many papers describe the extraction of biologically active compounds from seaweeds to obtain formulations useful in agriculture^1,10,17–21^ there is a little interest in the waste product, i.e., post-extraction residue, which may also be a potential raw material for industrial applications^4,14,22,23^. Management of this waste product complies with the principles of the circular economy, which is one of the components of sustainable^24^.

**Figure 1:**
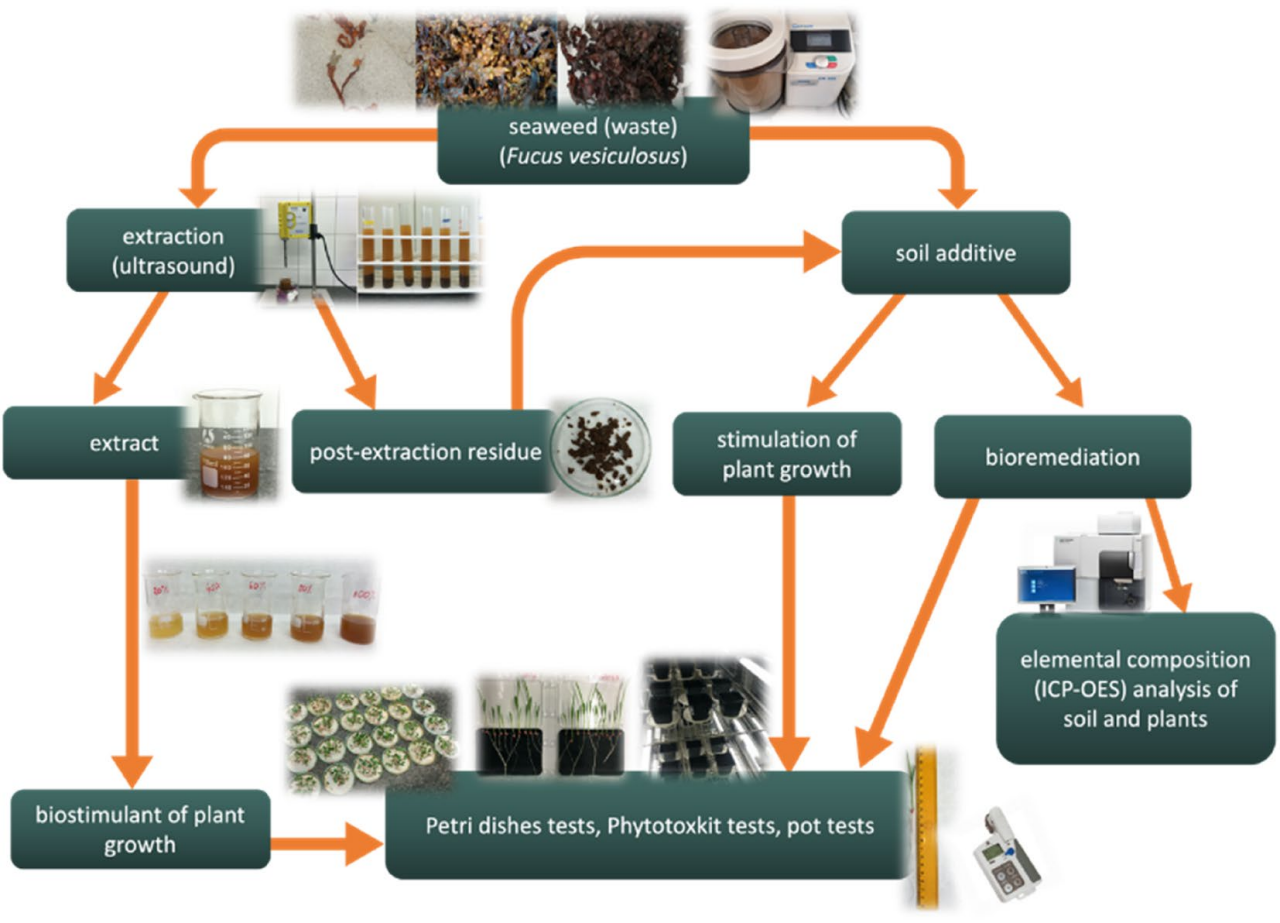
General scheme of the performed experiments.

The present work aimed at using the biomass of brown seaweed—*Fucus vesiculosus* to produce potential biostimulants for plant growth using ultrasound-assisted extraction (UAE). The biomass itself, as well as obtained post-extraction residue, were examined as soil amendments, which participated not only in the plant growth enhancement but also in the bioremediation of heavy metal ions in the soil through immobilization, reducing their mobility and availability to plants. The effectiveness of the proposed solutions was verified in plant germination tests to assess the potential phytotoxicity of *Fucus vesiculosus* extracts (biostimulation on radish (*Raphanus sativus*)) as well as in pot experiments (biostimulation on radish and bioremediation on sorgo (*Sorghum saccharatum*) seeds, i.e., seeds that are recommended for phytotoxkit tests). The general scheme of performed experiments is presented in Fig. 1.

## Materials and methods

### Chemicals

EMSURE ACS grade concentrated HNO_3_ (65%, m/v) and HCl (37% m/v) were obtained from Merck (Merck, KGaA, Darmstadt, Germany) and used for sample digestion. A commercially available Merck Certipur (Merck) multi-element stock (1000 mg/L) ICP standard solution (No. IV) and single ICP stocks (1000 mg/L) of As, Hg, P, S, and Se (all from Merck) were used for preparing standard solutions for calibration of the ICP OES instrument. To determine total phenolic compounds in *Fucus vesiculosus* extract Folin–Ciocalteu’s phenol reagent from Merck and gallic acid monohydrate from Sigma-Aldrich (Saint Louis, MI, USA) were applied. Zinc sulphate heptahydrate, necessary for Zn(II) solution preparation (case of bioremediation studies), was purchased from Avantor Performance Materials (Gliwice, Poland). Deionized water (18.3 MΏ cm) from an EASYpure RF water purification system (Barnstead Corp., USA) was used throughout.

### Collection and preparation of seaweeds

Fresh *Fucus vesiculosus* biomass was collected from the beach in Kolobrzeg (Poland) in June 2020. The tested algae are not collected from protected areas in Poland. The collected *F. vesiculosus* was identified by Izabela Michalak on the basis of its morphological characteristics in accordance with the taxonomic literature^25^. The biomass was washed with tap water to remove impurities, then air-dried, milled in Retsch GM 300 mill (4000 rpm/min), and finally sieved (500 µm sieve) to obtain a homogenous sample. The prepared seaweed biomass was used in the subsequent stages of the research.

### Ultrasound-assisted extraction of *Fucus vesiculosus*

Extract from *Fucus vesiculosus* was obtained via UAE using Hielscher UP100H ultrasonic homogenizer (Hielscher Ultrasonics, Teltow, Germany). For this purpose, 4.0 g of algal biomass was placed in a beaker containing 100 mL of water, homogenized for 30 min, and then centrifuged (Thermo Scientific™ Megafuge™ 40) at 4700 rpm for 10 min. The supernatant was treated as a 100% algal extract tested as a biostimulant of plant growth, while the separated post-extraction residue was dried and used as a potential soil additive for stimulation of plant growth or bioremediation of soil polluted with heavy metal ions.

### Evaluation of the utilitarian properties of seaweed bioproducts in tests on plants

For the evaluation of the utilitarian properties of *Fucus vesiculosus* bioproducts, several tests were performed. In order to examine the potential phytotoxicity of *Fucus vesiculosus* extract to plants, germination tests on radish seeds (*Raphanus sativus*) of the commercial variety ‘Pharaoh’ obtained from WerbAna Sp. z o.o. (Warsaw, Poland) for different concentrations of extract (E 20, E 40, E 60 E 80 and E 100%) were carried out. Preliminary soil studies, performed with the use of phytotoxkit tests (Tigret) and pots, allowed the assessment of the effect of seaweed and seaweed-derived products (extract and post-extraction residue) on sorgo growth. Sorgo seeds (*Sorghum saccharatum*) were purchased from TIGRET Sp. z o.o. (Warsaw, Poland). In addition, the phytotoxkit tests were also used to preliminarily examine the effectiveness of dry seaweed biomass/post-extraction residue in the bioremediation of soil contaminated with Zn(II) ions.

### Germination tests for seaweed extracts using radish (*Raphanus sativus*) seeds

Extract solutions of appropriate concentrations (20, 40, 60, and 80%) were prepared by diluting the 100% extract obtained by UAE with distilled water. The germination tests were performed on radish seeds in Petri dishes (diameter 85 mm). Simultaneously with each extract solution (N = 4), the control group (treated with distilled water) was prepared. Filter papers were laid out on each of the plates, and 25 radish seeds were placed at equal intervals. Then the seeds were evenly watered with 3 mL of the appropriate extract or distilled water. Plants were placed under a 57 cm high 45W LED Grow Light (GL-225RB-45W) for a week with a 12/12 h photoperiod and watered daily. After this time, the germination percentage was calculated. Next, the length of the roots, aboveground parts, and chlorophyll content in leaves was measured. Finally, the fresh mass of roots and aboveground parts was weighed for each of the dishes. Based on the statistical analysis of the obtained results, the concentrations of extracts that were non-toxic to plants and the best stimulating plant growth were selected.

### Phytotoxkit tests for sorghum seeds (*Sorghum saccharatum*) for selected extract concentrations, doses of seaweed, and post-extraction residue

For the phytotoxkit and pot tests, commercial soil (Athena—“universal potting soil”, Szczecinek, Poland) was used. According to the producer information, the pH of the soil produced based on peat, enriched with micro- and macroelements, was in the range of 5.5–6.5. The fresh soil was dried and subjected to the sieve analysis (2 mm sieve). Each phytotoxkit test contained 10 sorghum (*Sorghum saccharatum*) seeds, which were placed at equal intervals. The prepared tests were placed vertically under a 45W LED Grow Light (GL-225RB-45W) at a height of 57 cm, with a 12/12 h photoperiod. Phytotoxkit tests on sorghum seeds were performed in three variants:

1. *Effect of seaweed extract concentration* 20, 60, 80% (selection based on germination tests)—on the plant growth. For this purpose, 15 g of soil was mixed with 50 mL of distilled water (for the control group—C) or an extract with an appropriate concentration (for the experimental groups).
2. *Effect of soil additives* seaweed and post-extraction residue at doses of 2 and 4 g/kg (based on Alobwede et al.^26^)—on the plant growth. The soil was mixed evenly with algal additives.
3. *Effect of soil additives* seaweed and post-extraction residue at doses of 2 and 4 g/kg—on the plant growth in soil contaminated with Zn(II) ions. Before the experiments, the soil was evenly mixed with ZnSO_4_·7H_2_O solution. The content of Zn(II) ions in soil was assumed to be 1000 mg/kg, according to Chaoua et al.^27^. Then, appropriate soil additives were added. Additionally, in this variant, soil, as well as cultivated plants, were examined on the total concentration of Zn to assess Zn(II) ions accumulation.

All phytotoxkit tests lasted for 9 days and were performed in duplicate (N = 2). After the end of the tests, the roots length, the aboveground parts length, and the chlorophyll content of the individual plant were measured as well as the weight of aboveground parts for each phytotoxkit. Based on the statistical analysis of the obtained results, the extract and the dose of seaweed/post-extraction residue that best stimulated plant growth were selected for pot experiments.

### Pot tests with sorghum seeds (*Sorghum saccharatum*)

Pot tests on sorghum seeds (*Sorghum saccharatum*) were performed for soil with the addition of *Fucus vesiculosus* extract at the concentration of 20%, algal biomass of 2 g/kg, and post-extraction residue of 2 g/kg. Along with examined samples, the control one, without any additive, was prepared. In these pot tests, 50 mL of 20% extract or 0.12 g (2 g/kg) of seaweed biomass/post-extraction residue were added to 60 g of the soil and evenly mixed. Except that, 50 mL of distilled water was added to the soil with the addition of seaweed biomass/post-extraction residue. In each pot, 9 sorghum seeds were placed at equal intervals. The prepared pots were placed in a thermostatic cabinet with built-in lighting, a constant temperature of 20 °C, and a mean humidity of 86% (ST5C SMART, POL-EKO-APARATURA, Poland) for 17 days with a 12/12 h photoperiod. After this time, the length of the aboveground parts (hypocotyl and epicotyl), the chlorophyll content of individual plants as well as the mass of the aboveground parts for each group (pot) were measured. All pot tests were performed in three parallel samples (N = 3).

## Analytical methods

### pH and electrical conductivity of extract

The pH and electrical conductivity (EC) of seaweed extract were measured using a pH-meter with an additional conductivity measurement option (Mettler Toledo SevenCompact Duo, United States). All measurements were performed in duplicate (N = 2).

### Multi-element analysis of samples

Samples of the dry seaweed biomass, extract, dry post-extraction residue, dry soil, and dry parts of the plants (roots and leaves) were studied on the content of macro-, micro-, and trace elements by inductively coupled plasma-optical emission spectrometry (ICP-OES) method. Before spectrometric measurements, samples were prepared using microwave-assisted wet digestion in a closed-vessel system (Multiwave PRO; Anton Paar). Accordingly, about 5 g of a liquid sample or 0.25 g of a solid sample was weighed into Teflon vessels and poured with 5 mL of 65% (m/m) HNO_3_. In the case of soil samples, 5 mL of *aqua regia* (HCl:HNO_3_, 3:1, v/v) was used. Then the vessels were closed, placed in the rotor, and digested employing a 6-step microwave-assisted heating program with a maximum temperature of 190 °C for 60 min. Then, the sample remnants were transferred to polypropylene 30 mL containers and supplemented with deionized water to 25 g. All samples were prepared and analyzed in triplicate (N = 3). Blank samples were also prepared and included in the final results. Element concentrations were measured with an Agilent Model 5100 Dual View Dual View ICP spectrometer (Agilent, United States).

### Total phenolic compounds in *Fucus vesiculosus* extract

The total content of phenolic compounds in the extract of *Fucus vesiculosus* was determined using Folin–Ciocalteu reagent. In the procedure, to 0.5 mL of the seaweed extract, 2.5 mL of tenfold diluted Folin-Ciocâlteu reagent, and then 2 mL of 7.5% Na_2_CO_3_ solution were added. The prepared solution was heated in a water bath at 40 °C for 15 min with the next cooling for 5 min in an ice bath. The absorbance of the resulted mixture was measured at 765 nm using Spectrophotometer Biosens UV 5100 (Warsaw, Poland). The concentration of phenolic compounds was determined against the standard curve prepared for gallic acid and expressed as mg/L of gallic acid equivalents (GAE). The analyses were performed in triplicate (N = 3).

### Determination of chlorophyll content in cultivated plants

The relative chlorophyll content was measured in the plant leaves using a SPAD device (Konica Minolta, Japan). The values indicated by the meter are proportional to the chlorophyll content in the leaf. They were calculated based on the amount of radiation transmitted through the leaf in two ranges of radiation, differently absorbed by chlorophyll.

### Statistical analysis

Statistical analysis was performed using *Statistica* ver. 13.0 (TIBCO Software Inc., Tulsa, OK, USA). For all experimental groups, descriptive statistics (mean and standard deviations or median and quantiles) were performed. The Shapiro–Wilk test was used to assess the normality of the distribution of experimental results. The Brown-Forsythe’s test was used to check the homogeneity of variances. The statistical test (used to investigate the significance of differences between the tested groups) was selected based on the previously mentioned tests. The one-way analysis of variance (ANOVA) allowed the determination of the statistically significant differences between several groups. The Tukey multiple comparison test was used for normal distribution and homogeneous variances. For the lack of the normal distribution or lack of the homogeneity of variances, the Kruskal–Wallis test was used. The results were considered significantly different when *p* < 0.05.

## Results and discussion

### Characteristics of seaweed extract, dry seaweed biomass, and post-extraction residue

A brick-colored extract of *Fucus vesiculosus* was obtained using UAE. The extract pH was close to neutral (6.50 ± 0.02) and the mean EC value was 721.85 ± 3.75 μS/cm. Similarly, an almost neutral water extract of these algae (a pH range of 5.55–5.60) was obtained by Ferreira et al.^28^. Also, Sharma et al. received aqueous extracts from brown seaweeds, including *Fucus vesiculosus*, with a pH range close to this determined in the present study (5.21–6.30), but with a much higher EC value (9300 μS/cm)^19^. Significant differences in the electrical conductivity of aqueous extracts may result from the salinity of the water in which the extracted seaweeds were present. In the case of Strangford Lough (the east of Northern Ireland), the average water salinity is 33 PSU, while the waters of the Baltic Sea are only about 7 PSU.

A multi-element composition of the dry seaweed biomass, *Fucus vesiculosus* extract (at a concentration of 100%), and the post-extraction residue is shown in Table 1.

**Table 1:** Total concentrations of elements (mean ± SD, N = 3) in *Fucus vesiculosus* biomass (mg/kg dry weight), extract (mg/L) and post-extraction residue (mg/kg dry weight).

Element	Seaweed	Extract	Post-extraction residue
Al	574 ± 10	4.81 ± 0.09	474 ± 23
As	10.9 ± 0.1	< LOD	10.0 ± 0.5
B	99.1 ± 0.7	2.95 ± 0.11	86.8 ± 2.1
Ca	17,758 ± 13	2.93 ± 0.04	20,195 ± 412
Cd	0.687 ± 0.021	< LOD	0.755 ± 0.025
Cr	3.47 ± 0.13	< LOD	3.10 ± 0.10
Cu	1.62 ± 0.07	0.023 ± 0.001	1.63 ± 0.04
Fe	13.1 ± 0.5	4.36 ± 0.08	5.76 ± 0.20
Hg	< LOD	< LOD	< LOD
K	5271 ± 92	88.1 ± 1.3	4257 ± 169
Mg	7715 ± 43	29.6 ± 0.5	8258 ± 120
Mn	177 ± 1	1.08 ± 0.02	188 ± 2
Na	7244 ± 169	144 ± 2	5649 ± 180
Ni	4.82 ± 0.11	0.112 ± 0.004	4.67 ± 0.07
P	668 ± 10	10.3 ± 0.2	536 ± 36
Pb	0.950 ± 0.102	< LOD	0.865 ± 0.111
S	23,955 ± 99	107 ± 2	27,340 ± 855
Se	< LOD	< LOD	< LOD
Zn	46.8 ± 0.6	0.486 ± 0.006	55.7 ± 1.8

*< LOD* – below the detection limit, *SD* – standard deviation.

Seaweed biomass contained mainly Ca (1.78%), K (0.53%), Mg (0.77%), Na (0.72%), P (0.067%), and S (2.40%), i.e., important macroelements for the proper plant growth and development^29^. Similarly, Sharma et al. determined high content of Na (3.70%), S (3.13%), K (3.04%), and Ca (1.41%) in dry *Fucus vesiculosus* collected from Strangford Lough (Northern Ireland)^19^. Also, the content of S in the examined *Fucus* corresponded well with the results obtained by Bikovens et al. (2.30%) for dry *Fucus vesiculosus* biomass (collected as a waste from the Gulf of Riga)^4^. The range of the mineral content in *Fucus vesiculosus* determined in our study also agreed with the report of Balina et al., who showed that this seaweed collected from the Gulf of Riga (Latvia) contained mainly S (2.82%), Ca (2.15%), K (1.10%), Mg (0.93%) and P (0.14%)^6^. In light of this, it can be concluded that the element composition of *Fucus vesiculosus* may be advantageous for agricultural use. The recycling of the algal biomass back to agricultural land as soil additives/amendments, to improve soil quality and crop nutrition, could be one of the elements of circular economy fertilization^26^.

The analysis of the element composition of the extract showed that it consisted mainly of Na, S, and K (88–144 mg/L). Quite high values for Mg, P, Al, and Fe were also obtained (4–30 mg/L). These elements may be responsible for the potential stimulation of plant growth after soil or foliar application of *Fucus vesiculosus* extract. Noteworthy, the toxic metal ions such as As, Cd, Cr, Hg, and Pb were not extracted from the biomass (their concentration in the extract was below the detection limit), which proves that the *Fucus vesiculosus* extract is safe and can be used as a potential plant growth biostimulant. Its usage will not carry the risk of soil or plant contamination with toxic metals.

Valuable element composition was also obtained for the *Fucus vesiculosus* post-extraction residue. As shown in Table 1, despite the sample extraction, it still contained valuable both, micro- and macroelements, especially S, Ca, Mg, K and P. Therefore, it creates a potential for the management of this waste product in the process of plant growth biostimulation or bioremediation of polluted soil. Valorization of this waste into components of fertilizers with microelements as an element of the biorefinery concept is proposed^4,30^.

It was also found that the examined *Fucus vesiculosus* extract contained polyphenols, and the total concentration of phenolic compounds was 0.530 ± 0.009 mg GAE/L. It corresponded to 13.3 ± 0.2 mg GAE per 1 g of dry seaweed biomass used in the extraction process. Our results are nearly two times lower than that obtained by Díaz-Rubio et al. for a commercial *Fucus vesiculosus* extract (24.5 mg/g dry weight)^31^. Phenolic compounds present in the seaweed extract, due to their antioxidant properties, may be responsible for the increase in plant resistance to biotic (e.g., bacteria, fungi, viruses, insects) and abiotic stress (e.g., UV radiation, low temperature, high salinity)^9^.

### Phytotoxicity tests of seaweed extracts using radish (*Raphanus sativus*) seeds

Germination tests with the use of radish seeds allowed us to check the potential phytotoxicity of examined extracts and to select the concentration of *Fucus vesiculosus* extract that will best stimulate plant growth. The results of the measured parameters of *Raphanus sativus* plants (length and weight of roots and aboveground parts of the plant, chlorophyll content) for different concentrations of *Fucus vesiculosus* extract were detailed in Table 2. The results for the control group (without the extract) were also included in this table. Mean and standard deviation (SD) were used in the case of normal distribution of the obtained results, whereas the median for the distribution other than normal was applied.

**Table 2:** The results of germination tests on radish seeds treated with various concentrations of seaweed extract.

Parameter	Root length (cm)	Roots weight (g)	Aboveground part length (cm)	Aboveground part weight (g)	Chlorophyll content—SPAD Index (–)
Group	Median	Mean ± SD	Median	Mean ± SD	Mean ± SD
E (20%)	6.4^a,b^ (N = 90)	0.231 ± 0.077 (N = 4)	3.1 (N = 90)	1.28 ± 0.20 (N = 4)	55.7 ± 8.2 (N = 81)
E (40%)	5.3^c,d^ (N = 72)	0.180 ± 0.037 (N = 4)	3.0^a^ (N = 72)	1.03 ± 0.42 (N = 4)	54.5 ± 9.6 (N = 65)
E (60%)	5.6^e^ (N = 86)	0.198 ± 0.035 (N = 4)	3.3 (N = 86)	1.39 ± 0.22 (N = 4)	55.7 ± 9.4 (N = 71)
E (80%)	7.1^c,f,g^ (N = 78)	0.236 ± 0.041 (N = 4)	3.6^a,b^ (N = 78)	1.42 ± 0.28 (N = 4)	52.9 ± 11.3 (N = 63)
E (100%)	4.2^a,f^ (N = 71)	0.176 ± 0.067	3.0 (N = 71)	1.05 ± 0.34 (N = 4)	52.5 ± 12.7 (N = 55)
C (H_2_O)	3.5^b,d,e,g^ (N = 62)	0.175 ± 0.039 (N = 4)	3.1^b^ (N = 62)	1.24 ± 0.23 (N = 4)	54.4 ± 11.9 (N = 57)

*N* number of measurements in each group, *E* experimental group (extract concentration in the bracket), *C* control group.

^a,b,c…^Statistically significant differences for *p* < 0.05 (Kruskal–Wallis test; results present as a median).

Germination tests showed that there was no phytotoxic effect of the extracts on the radish growth. The stimulation of plant growth was demonstrated for all tested extract concentrations. However, the outcome of this study did not give unambiguous results, which extract concentration stimulated the initial phase of radish growth best. In general, the value of the measured parameters (except chlorophyll) increased with the increase in the concentration of the extract to 80%. The weakest stimulating effect was observed for the highest concentration of the extract (100%), which is a concentrate of biologically active compounds. It is recommended to dilute such extracts before applying to plants as they can damage the plants. According to Cruch and van Staden, seaweed extracts are bioactive at low doses and should be diluted as 1:1000 or even more^32^.

According to the statistical analysis, various concentrations of *Fucus vesiculosus* extracts influenced radish growth. The median root length in all experimental groups was greater than in the control group, which shows the stimulating effect of *Fucus vesiculosus* extract on the radish root growth. Statistically significant differences were found between E 20% and E 100%, between E 20% and the control group, successively between E 40% and E 80%, and between E 40% and the control group. In addition, statistically significant differences were also found between E 60% and the control group, between E 80% and E 100%, and between E 80% and the control group. Compared to the control group, the highest root length (7.1 cm) was obtained for E 80% and it was more than twice as long (103% more than in the control group). A slightly lower median root length was demonstrated in the group with 20% extract (an increase of 83% compared to the control group) and with 60% extract (an increase of 60% compared to the control group).

Similarly, the mean root weight in all experimental groups was higher than in the control group, proving the stimulating properties of tested seaweed extract on plant growth. The highest weight (0.236 g) was achieved in the group treated with 80% extract, which was 35% higher than in the control group. A slightly lower result was achieved in the group with 20% extract (an increase of 32% compared to the control group).

The median aboveground length of the plant was greater for the 60% and 80% extracts than in the control group. In the group treated with 80% extract, the highest value among the studied groups was achieved (3.6 cm) and it was 16% higher than in the control group. In the remaining groups (20, 40, and 100% extracts), the median length of the aboveground part was almost equal (20%) or lower (40 and 100%) than this of the control group.

Except for 40 and 100% extracts, the mean weight of the aboveground parts was higher in the experimental groups than in the control group. The highest mean weight of plant roots (1.42 g) was obtained in the group with 80% extract and it was 14% higher than in the control group. In the group with 60% extract this value was slightly lower, but still 12% higher than in the control group.

In the case of the chlorophyll content, the results were similar in all tested groups. Higher values than in the control group were obtained for three experimental groups, i.e., with 20, 40, and 60% extract. The highest chlorophyll content (55.7) was determined in the groups treated with 20 and 60% extract. These results were only 2.4% higher than in the control group, so the tested seaweed extracts did not significantly increase the chlorophyll content in radish during its growth.

Based on germination tests performed in this study, for the next step—phytotoxkit tests on soil—the following extract concentrations were selected: 20% (due to the high content of chlorophyll in the seedlings), 60%, and 80% (the greatest impact on the biometric parameters of radish seedlings).

Concluding, germination tests on radish showed that different concentrations of *Fucus vesiculosus* extract stimulated the growth of this plant. Our results coincide with those presented by the others. As an example, extracts from *Fucus vesiculosus* and *Fucus serratus* stimulated the growth (dry matter yield, percentage of dry matter, and a number of roots) of mung bean (*Vigno mungo*) and pak choi cabbage (*Brassica rapa chinensis*)^19^, whereas *Fucus spiralis* extract positively influenced the growth of common bean (*Phaseolus vulgaris*) in terms of the length of the aboveground part and root as well as chlorophyll *a* and *b* contents^21^. As an essential photosynthetic pigment for plants, chlorophyll is mainly responsible for photosynthesis and, consequently for the growth and development of plants. The degree of plant productivity is directly correlated with the photosynthetic capacity of leaves^33^. The positive effect of *Fucus vesiculosus* extracts on plant growth could be attributed to the chemical composition of the seaweed extract (plant growth hormones, nutrients, or vitamins)^21^ and improved uptake of nutrients by plants^19^. According to Bikovens et al., the positive effect of the application of *Fucus vesiculosus* extract on plant growth (*Avena sativa*) resulted from the content of polysaccharides – e.g., fucoidan and phenolic compounds with antioxidant properties^4^.

### Phytotoxkit tests with sorghum (*Sorghum saccharatum*) seeds for selected extract concentrations

Phytotoxkit tests on soil conditions during the cultivation of sorghum (*Sorghum saccharatum*) seeds at selected concentrations of *Fucus vesiculosus* extract (20, 60, and 80%) were performed. These tests to a greater extent reflect the research conducted in greenhouse conditions and will allow to verify the results obtained in the germination tests regarding the selection of the *Fucus vesiculosus* extract concentration. The results of the measured parameters such as the length of root and aboveground part as well as chlorophyll content of sorghum grown in the both experimental and control group are shown in Table 3.

**Table 3:** Results (expressed as median or mean ± SD) of phytotoxkit tests on sorghum (*Sorghum saccharatum*) seeds for selected concentrations of seaweed extracts.

Parameter	Root length (cm)	Length of aboveground part (cm)	Weight of aboveground parts (g)	Chlorophyll content—SPAD Index (–)
Group	Median	Mean ± SD	Mean ± SD	Mean ± SD
E (20%)	11.3^a^ (N = 19)	7.7 ± 2.9 (N = 19)	1.07 ± 0.06 (N = 2)	27.7 ± 4.0 (N = 19)
E (60%)	9.6 (N = 17)	6.4 ± 3.5 (N = 17)	0.718 ± 0.029 (N = 2)	25.6 ± 4.4 (N = 17)
E (80%)	10.8 (N = 20)	6.8 ± 2.7 (N = 20)	1.08 ± 0.04 (N = 2)	26.5 ± 3.2 (N = 20)
C (H_2_O)	9.9^a^ (N = 18)	6.7 ± 3.4 (N = 18)	0.657 ± 0.006 (N = 2)	25.0 ± 4.3 (N = 18)

*N* number of measurements in each group, *E* experimental group (extract concentration in the bracket), *C* control group.

^a^Statistically significant differences for *p* < 0.05 (Kruskal–Wallis test; results present as a median).

In the case of the root length, the result higher than in the control group was obtained for the 20 and 80% extract. A statistically significant difference occurred between the control group and the 20% extract, for which the highest value of root length (11.3 cm) was measured.

Also, for 20% extract, the highest length of the aboveground part of the plant (7.7 cm) was obtained. There were no statistically significant differences between the examined groups, but the plant length for 20% extract was higher by 15% than in the control group. A higher value of the mean aboveground part length than in the control group was also observed for 80% extract.

The mean weight of the aboveground parts was higher for all studied groups than for the control group. The highest result was obtained in the group treated with 80% extract (an increase of 64% compared to the control group). Similarly, a high result was obtained also in the group with 20% extract.

For all experimental groups, the mean content of chlorophyll was higher than in the control group, but these differences were not statistically significant. The highest value was achieved for 20% extract (an increase of 11% in comparison to the control group).

Based on conducted experiments, it can be concluded that among the tested concentrations of *Fucus vesiculosus* extract, the lowest one, i.e., 20% stimulated the best growth of sorghum, both in terms of the root length, the aboveground part, and the chlorophyll content. This is desirable from an economic point of view—from the final concentration of the extract (100%) obtained by UAE, much larger volumes of the desired formulation with a concentration of 20% can be obtained.

A survey of literature confirms that lower concentrations of algal extracts have a positive effect on plant growth^17,32^. Latique et al. studied the effect of *Fucus spiralis* extract, prepared by boiling in distilled water, on the growth of common bean (*Phaseolus vulgaris*). From all studied seaweed extract concentrations (12.5, 25, 50, and 75%), the greatest length of the aboveground plant part and the root (an increase of 67%) and the values of chlorophyll *a* and *b* (an increase of 200%) for 25% extract were obtained^21^. Also, El Kaoaua et al. during the study of the effect of different *Fucus spiralis* extract concentrations (12.5, 25, and 50%) on sage (*Salvia officinalis*) growth observed that the highest plant length was for 25% extract, for which total chlorophyll content was almost 2-times higher than in the control group^34^. The positive response in plant growth for seaweed extracts may be related to the presence of micro- and macroelements, amino acids, hormones (e.g., cytokinins, auxins), and vitamins in their biomass^21,33^.

### Phytotoxkit tests on sorghum (*Sorghum saccharatum*) seeds in soil supplemented with dry seaweed biomass and post-extraction residue

In the next stage, the effect of dry seaweed biomass and post-extraction residue, applied at doses of 2 and 4 g/kg to the soil, on sorghum (*Sorghum saccharatum*) seeds growth was examined and the results (as median or mean ± SD) are collected in Table 4.

**Table 4:** Results of phytotoxkit tests on sorghum (*Sorghum saccharatum*) seeds cultivated in soil supplemented with dry seaweed and its post-extraction residue.

Parameter	Root length (cm)	Length of aboveground part (cm)	Weight of aboveground parts (g)	Chlorophyll content—SPAD Index (–)
Group	Median	Median	Mean ± SD	Mean ± SD
Post-extraction residue (2 g/kg)	11.0^a,b,c^ (N = 19)	4.5 (N = 19)	0.397 ± 0.014 (N = 2)	25.9 ± 3.2 (N = 16)
Post-extraction residue (4 g/kg)	9.5 (N = 19)	4.3 (N = 19)	0.313 ± 0.123 (N = 2)	25.7 ± 2.2 (N = 14)
Seaweed (2 g/kg)	9.1^a^ (N = 20)	4.1 (N = 20)	0.363 ± 0.125 (N = 2)	25.3 ± 3.5 (N = 15)
Seaweed (4 g/kg)	8.9^b^ (N = 20)	3.8 (N = 20)	0.272 ± 0.036 (N = 2)	27.6 ± 2.9 (N = 12)
C (H_2_O)	8.1^c^ (N = 19)	4.5 (N = 19)	0.277 ± 0.025 (N = 2)	25.5 ± 2.4 (N = 9)

*N* number of measurements in each group, *C* control group.

^a,b,c^Statistically significant differences for *p* < 0.05 (Kruskal–Wallis test; results present as a median).

Both, for *Fucus vesiculosus* itself and for the post-extraction residue, better results (in terms of length of root and aboveground part, as well as the weight of the aboveground part) were obtained for the lower dose of soil additive, i.e., 2 g/kg.

The median root length in each experimental group was higher than for the control group, which shows the positive effect of the studied soil additives on this parameter. Statistically significant differences were found between the group with the 2 g/kg of post-extraction residue and the control group, the group with seaweeds at a dose of 2 g/kg and seaweeds at a dose of 4 g/kg.

Considering the median of the aboveground length, no major differences between the groups were observed. In none of the experimental groups, this value was higher than in the control group. In the case of the group with the dose of 2 g/kg post-extraction residue, the result was equal to that of the control (4.5 cm), which is also the highest result among all the tested groups.

On the other hand, an increase in the mean weight of the aboveground parts was observed for the experimental groups with post-extraction residue at two tested doses (2 and 4 g/kg) and seaweed biomass at a dose of 2 g/kg. The highest value was obtained in the group with the post-extraction residue at a dose of 2 g/kg and although no statistically significant differences were found, this value (0.397 g) was 43% higher than in the control group. The mean weight of the aboveground part in the group with seaweed biomass at a dose of 2 g/kg was 31% higher, while the result for the group with the post-extraction residue at a dose of 4 g/kg was enhanced by 13% if compared with the values for the control group.

The mean content of chlorophyll was slightly higher in the groups with the post-extraction residue (2 and 4 g/kg) as compared to the control group. In the group with seaweed biomass at a dose of 2 g/kg this value was slightly lower than in the control group. On the other hand, the highest value (27.6) was obtained in the group with seaweed biomass at a dose of 4 g/kg and it was 8.2% higher than in the control group. Thus, the addition of dry seaweed biomass to the soil at a dose of 4 g/kg caused a greater increase in chlorophyll content in sorghum than in other experimental groups, but still, the change was insignificant.

Generally, our results coincide with literature data on the use of seaweed-derived products as soil amendments. Accordingly, Bikovens et al. compared the effect of the soil addition of *Fucus vesiculosus* and its post-extraction residue at doses of 5 and 10 g/kg on the growth of oat (*Avena sativa*) and found that seaweed biomass at higher dose stimulated the best root length and count of tips, whereas no effect of experimental groups on average diameter or root volume was observed. Additionally, the authors showed that the post-extraction residue after isolation of fucoidan and phenolic compounds with biological and antioxidant activities is still a valuable material to be used as a fertilizer additive in agriculture^4^. Ahmed et al. proved that seaweeds—*Ulva fasciata*, *Sargassum lacerifolium*, and their mixture can be used not only for the stimulation of radish (*Raphanus sativ*us) growth but also for bioremediation of soil contaminated with heavy metals. In the cited work, the addition of 10 g of seaweed biomass to 40 g of soil not only increased the germination percentage, root and shoot length, and weight as well as leaf area, but also improved the content of organic matter and nutrients in the soil. The best results in terms of the measured parameters were obtained for the mixture of seaweeds, which provided plants with nutrients and phytohormones like gibberellins, cytokinins and indole acetic acid^13^. The stimulating effect of dry seaweed soil additive on tomato (*Solanum lycopersicum*) growth was also obtained by Salcedo et al.^35^. The supplementation of *Undaria pinnatifida* at doses of 0.1 and 1% improved organic matter content in the soil as well as the growth parameters such as aboveground parts (fresh and dry weight), roots dry weight, leaf area, and total chlorophyll content as compared to the control group. This effect was explained by the chemical composition of *Undaria* biomass, i.e., the presence of plant nutrients such as minerals, vitamins, and phytohormones—cytokinin, gibberellin, and auxin^35^. As shown in the research of El-Katony et al., brown seaweed *Dictyota dichotoma* used as an organic soil amendment improved the rice (*Oryza sativa*) productivity by enriching the soil with plant macro- and microelements and bioactive compounds (e.g., growth hormones, vitamins, phenolics, flavonoids, fucoidan), increasing the plant resistance to the abiotic stress (salt and water stress) as well as improving the soil structure and capacity to hold the nutrients and water^36,37^. Possinger and Amador obtained equivalent or improved yield and quality of sweet corn (*Zea mays*) after adding seaweed dried biomass in two contents: ~ 10,910 kg/dry weight/ha and ~ 21,820 kg dry weight/ha, as compared to preformulated organic fertilizer treatment. They explained these results with improved soil quality and increased nutrient supply due to the addition of seaweed amendments^38^. Due to the increasing costs of inorganic fertilizers, the use of seaweeds, an inexpensive and nutrient-rich resource, may provide an alternative to improving soil fertility, especially in coastal areas. *Fucus vesiculosus* studied in this paper, which is particularly rich in minerals (as shown in Table 1), can be a valuable addition to the soil for modern agriculture, especially in organic farming systems.

### Pot tests with sorghum (*Sorghum saccharatum*) seeds

To confirm the effectiveness of *Fucus vesiculosus*-derived products—dry biomass, post-extraction residue, and extract, and to compare their effect on sorghum (*Sorghum saccharatum*) seeds growth, pot experiments were performed for the best doses determined in previous steps. The results of the measured parameters (hypocotyl and epicotyl length, the entire aboveground part length, aboveground parts mass, chlorophyll content) for the studied experimental groups—dry seaweed biomass at a dose of 2 g/kg, the post-extraction residue at a dose of 2 g/kg and 20% extract as soil additive are listed in Table 5. Generally, for all measured parameters, the obtained values were higher in experimental groups as compared to the control group. Thus, these results confirm the biostimulation of plant growth using dry seaweed biomass (2 g/kg), post-extraction residue (2 g/kg), and 20% *Fucus vesiculosus* extract during its cultivation.

**Table 5:** Results (median or mean ± SD) of pot tests with the use of sorghum (*Sorghum saccharatum*) seeds (after 17 days).

Parameter	Hypocotyl length (cm)	Epicotyl length (cm)	Length of entire aboveground part (cm)	Weight of aboveground parts (g)	Chlorophyll content—SPAD Index (-)
Group	Median	Median	Median	Mean ± SD	Median
Post-extraction residue (2 g/kg)	4.7^b,c^ (N = 27)	13.4^b^ (N = 27)	18.2^b^ (N = 27)	1.13 ± 0.09^b’^ (N = 3)	27.0 (N = 27)
Seaweed (2 g/kg)	4.6^a^ (N = 26)	13.2^a^ (N = 26)	17.8^a^ (N = 26)	1.03 ± 0.16^a’^ (N = 3)	26.2 (N = 26)
E (20%)	5.0^b,d^ (N = 25)	13.4^c^ (N = 25)	18.7^c^ (N = 25)	1.12 ± 0.10^c’^ (N = 3)	27.4 (N = 25)
C (H_2_O)	4.2^a,c,d^ (N = 23)	11.4^a,b,c^ (N = 23)	15.6^a,b,c^ (N = 23)	0.681 ± 0.168^a’,b’,c’^ (N = 3)	26.4 (N = 23)

*N* number of measurements in each group, *E* experimental group (extract concentration in the bracket), *C* control group.

^a,b,c,d^Statistically significant differences for *p* < 0.05 (Kruskal–Wallis test; results present as a median).

^a’,b’,c’^Statistically significant differences for *p* < 0.05 (Tukey's test; results present as a mean).

The longest hypocotyl length was obtained in the group treated with 20% extract (an increase of 19%). The longest epicotyl length (an 18% increase of 18%) was obtained simultaneously in the group with the post-extraction residue and the extract. Finally, the longest length of the entire aboveground part (an increase of 20%) was obtained in the group with the extract. Statistically significant differences were found between all experimental groups and the control group (in the case of measuring the length of the hypocotyl, epicotyl, and the whole aboveground part) and additionally for the hypocotyl length between the group with the post-extraction residue and the extract.

The highest weight of the aboveground part was obtained in the group with the post-extraction residue (value higher by 66% than in the control group), but this result slightly differed from the result in the group with the extract. Also, statistically significant differences were found between all experimental groups and the control group.

Taking into account the chlorophyll content, no statistically significant differences between the determined values in all the tested groups were found. The highest result was obtained in the group with 20% extract; however, it was only 4% higher than in the control group.

All three tested soil additives obtained from *Fucus vesiculosus* stimulated the growth of sorghum seeds, but the best effect (hypocotyl and epicotyl length, the entire aboveground part length, chlorophyll content) was obtained after the use of 20% extract. It can be assumed that the biologically active ingredients contained in the extract were faster and easier absorbed by plants than in the case of the soil additive. Similar results for the pot cultivation of rice (*Oryza sativa*) were presented by El-Katony et al.^36^. Greater improvement in rice growth (in terms of grain yield, number of grains/spike, number of spikes/plant, and seed index) was noted for algal aqueous extract (prepared from brown seaweed *Dictyota dichotoma*) than for algal powder applied directly to the soil.

### Phytotoxkit tests with sorghum (*Sorghum saccharatum*) seeds on soil contaminated with Zn(II) ions

So far, many studies have been conducted on the removal of heavy metal ions by seaweeds (including *Fucus* sp.) from polluted water, but only a few of them concern the removal of heavy metals from contaminated soil. Therefore, preliminary studies were carried out to assess the possibility of using algal biomass and post-extraction residues in the bioremediation of soil contaminated with toxic metal ions via their bioimmobilization. Organic additives in soil are known to be able to immobilize/stabilize heavy metal ions and reduce their availability to plants^13,16,39,40^. The effect of the addition of dry *Fucus vesiculosus* and its post-extraction residue (at 2 and 4 g/kg doses) to the soil contaminated with Zn(II) ions (1000 mg/kg) on the early plant growth and accumulation of zinc in the aboveground part of sorghum was investigated. Table 6 shows the results of the measured parameters of sorghum seeds (root and aboveground part length, weight of the aboveground parts, chlorophyll content) for the experimental groups and two control groups (uncontaminated soil and contaminated soil, both without the addition of seaweed biomass and post-extraction residue). All the results (except those for the mass of the aboveground part of seeds) were expressed as median; in the case of the mass parameter the mean along with SD was applied.

**Table 6:** Results of phytotoxkit tests with sorghum seeds on soil contaminated with Zn(II) ions with the addition of dry seaweed biomass and post-extraction residue.

Parameter	Root length (cm)	Length of aboveground part (cm)	Weight of aboveground part (g)	Chlorophyll content—SPAD Index (–)
Group	Median	Median	Mean ± SD	Median
Contaminated soil with Zn(II) ions + post-extraction residue (2 g/kg)	8.2^a,b,c,d^ (N = 19)	8.0 (N = 19)	0.379 ± 0.116 (N = 2)	30.3 (N = 13)
Contaminated soil with Zn(II) ions + post-extraction residue (4 g/kg)	12.6^b^ (N = 20)	8.0 (N = 20)	0.570 ± 0.021 (N = 2)	28.4 (N = 17)
Contaminated soil with Zn(II) ions + seaweed (2 g/kg)	11.0^a^ (N = 19)	10.5 (N = 19)	0.575 ± 0.047 (N = 2)	29.8^a^ (N = 17)
Contaminated soil with Zn(II) ions + seaweed (4 g/kg)	10.1 (N = 19)	7.7 (N = 19)	0.539 ± 0.180 (N = 2)	29.1 (N = 17)
C (soil contaminated with Zn(II) ions)	11.1^d^ (N = 20)	10.8 (N = 20)	0.705 ± 0.056 (N = 2)	28.2 (N = 18)
C (uncontaminated soil)	11.1^c^ (N = 20)	8.4 (N = 20)	0.546 ± 0.023 (N = 2)	27.6^a^ (N = 19)

*N* number of measurements in each group, *C* control group.

^a,b,c…^Statistically significant differences for* p* < 0.05 (Kruskal–Wallis test; results present as a median).

No phytotoxic effect of Zn(II) ions on plants was demonstrated when the soil was contaminated with this toxic metal above the allowed limit proposed by Chaoua et al.^27^. The stimulating effect of Zn(II) ions on the growth of sorghum (an increase in length and weight of the aboveground part, chlorophyll content) was obtained for the control group cultivated on the contaminated soil (phytoremediation process). For the examined soil additives applied at both doses to the contaminated soil, a decrease in the length of root and aboveground part was observed as compared with the control group (cultivated on contaminated soil). Such effect could be due to the reduced bioavailability of zinc to plants, which is known to be involved in various structural and/or catalytic functions like cell division, cell expansion and protein synthesis, and is a cofactor for several enzymes or a constituent of metalloenzyme^41^. *Fucus vesiculosus* and the post-extraction residue could act as agents immobilizing zinc ions in the soil.

Again, it was shown that the dose of 2 g/kg of algal biomass was better than 4 g/kg (higher values for all tested parameters were obtained). In the case of the post-extraction residue, better results were obtained for the dose of 4 g/kg (except for the chlorophyll content). Comparing the two tested control groups, it can be concluded that in the case of length and weight of the aboveground parts and the chlorophyll content, higher values were obtained in the control group with the contaminated soil. It can indicate the stimulating properties of Zn(II) ions. In contrast, root length was equal in both groups.

To check the bioaccumulation of Zn(II) ions in the aboveground part of sorghum grown in the contaminated soil with and without soil amendment, its content in the soil before bioremediation as well as in the aboveground part plant biomass was determined. The results are presented in Table 7. Additionally, for each examined group, the bioaccumulation factor (BF), describing the ability of plants to accumulate elements from the substrate (soil), was calculated and included in this table. The BF factor (in %) referred to the ratio of the metal content in the plant to its content in the soil.

**Table 7:** The total content of Zn (mean ± SD, N = 2 in mg/kg dry weight) in soil (before bioremediation) and in aboveground sorghum parts after bioremediation.

Group	Soil before bioremediation	Aboveground sorghum parts	BF (%)
Contaminated soil with Zn(II) ions + seaweed 2 g/kg	756 ± 2	129 ± 2	17.1%
Contaminated soil with Zn(II) ions + seaweed 4 g/kg	746 ± 8	114 ± 1	15.3%
Contaminated soil with Zn(II) ions + post-extraction residue 2 g/kg	780 ± 4	75.8 ± 0.6	9.72%
Contaminated soil with Zn(II) ions + post-extraction residue 4 g/kg	774 ± 5	122 ± 1	15.8%
C (uncontaminated soil)	8.76 ± 0.08	41.2 ± 0.2	–
C (soil contaminated with Zn(II) ions)	781 ± 4	146 ± 1	18.7%

*C* control group, *BF* bioaccumulation factor.

The addition of *Fucus vesiculosus*-derived soil amendments decreased the bioaccumulation of zinc in sorghum from the tested experimental groups. The lowest BF in the aboveground parts of sorghum was calculated for the post-extraction residue applied at a dose of 2 g/kg and it was almost two times lower than in the control group with soil contaminated with Zn(II) ions, where phytoremediation occurred. This means that organic soil amendments can act as metal ions sorbents in the soil, reducing their availability to plants^11,13,16,39,40^. It is worth mentioning that *Fucus vesiculosus* is an effective biosorbent for the uptake of Zn(II) ions from wastewater. 66% of the dry weight of seaweed are polysaccharides, which are believed to be responsible for the uptake of metals. Due to the presence of functional groups on the surface of biomass (e.g., carboxyl, hydroxyl, amino), this seaweed can bind metal ions^42^.

The post-extraction residue may have better biosorption properties than dry seaweed biomass due to the extraction process that disrupts the biomass to isolate active compounds. This conclusion indicates another way of managing the waste product from the extraction process, following the principles of sustainable development and circular economy. In addition to biostimulation of plant growth, the post-extraction residue can also be used to bioremediate soil from toxic metals such as Zn. Abd-Elhady obtained similar results in a study of lettuce (*Lactuca sativa*) grown in soil also contaminated with zinc ions. When dry seaweed biomass was added to the soil, the total uptake of Zn(II) ions by the roots was about 12% higher, while their accumulation in the aboveground parts decreased by about 26%^11^. Also, Ahmed et al. showed that the biosorption of heavy metals in the soil by seaweeds (*Ulva fasciata* and *Sargassum lacerifolium*) reduced their bioaccumulation in the roots of radish plants and translocation to shoots and additionally can boost the plant tolerance to the heavy metal stress^13^.

## Conclusions

The conducted research indicates three possibilities for the management of seaweed *Fucus vesiculosus* into products useful for agriculture and a clean environment. A series of plant tests were carried out to assess the effect of *Fucus vesiculosus*-derived products (dry biomass/extract/post-extraction residue) on early plant growth. The application of an environmentally friendly technique – ultrasound-assisted extraction allowed the production of extract from the seaweed biomass. In the germination tests, the extract concentration, which was not phytotoxic to plants and stimulated plant growth was selected. Phytotoxkit and pot tests proved that not only algal extracts (especially at concentration of 20%) but also seaweed biomass as well as post-extraction residue (especially at a dose of 2 g/kg) used as soil additives can positively influence the plant growth. The biostimulating properties of *Fucus vesiculosus* extract can be attributed to the content of micro- and macroelements that are crucial for plant development. We showed that *Fucus vesiculosus* biomass can play a dual role—as a soil amendment (on the one side) and as heavy metals bioremediation agent in the soil through their bioimmobilization (on the other side). These results demonstrate the enormous potential of waste biomass management and by-products obtained during its processing. This approach is in line with the principles of the circular economy, which is the path to sustainable development and helps to protect the environment.

## Data Availability

All relevant data are within the paper.
